# Temporal trends of contaminants in Arctic human populations

**DOI:** 10.1007/s11356-018-2936-8

**Published:** 2018-08-25

**Authors:** Khaled Abass, Anastasia Emelyanova, Arja Rautio

**Affiliations:** 1 Arctic Health, Faculty of Medicine, University of Oulu, P.O. Box 5000, FI-90014 Oulu, Finland; 2 Department of Pesticides, Menoufia University, P.O. Box 32511, Menoufia, Egypt; 3 Thule Institute & University of Arctic, University of Oulu, Oulu, Finland

**Keywords:** Contaminants, POPs, Arctic, Children, Breast milk, Maternal blood, Trend analysis, Health outcomes, Russian Arctic

## Abstract

The first Arctic Monitoring and Assessment Programme (AMAP) report was published in 1998 and followed by three assessment reports of human health (AMAP 2003, 2009 and 2015). The focus area of the AMAP reports was to monitor levels of environmental contaminants in the Arctic and to assess the health effects connected with detected levels in Arctic countries. This review gives an overview of temporal trends of contaminants and their health effects in humans of the Arctic based on data published by AMAP, as well as Russian scientific literature. Several time series of 31 contaminants in humans of the Arctic from different cohorts are reported. The lengths of time series and periods covered differ from each other. International restrictions have decreased the levels of most persistent organic pollutants in humans and food webs. Percentage changes for contaminants in human biological matrices (blood samples from children, mothers and males and breast milk samples) for the period of sampling showed declining trends in most of the monitored Arctic locations, with the exception of oxychlordane, hexachlorobenzene (HCB), 2,2′,4,4′,5,5′-hexabromodiphenyl ether (PBDE153) and perfluorinated compounds (PFCs).

**Electronic supplementary material:**

The online version of this article (10.1007/s11356-018-2936-8) contains supplementary material, which is available to authorized users.

## Introduction

The Arctic monitoring and assessment programme (AMAP), an Arctic Council working group, was established in the early 1990s. The AMAP mission is ‘*providing reliable and sufficient information on the status of, and threats to, the Arctic environment, and providing scientific advice on actions to be taken in order to support Arctic governments in their efforts to take remedial and preventive actions relating to contaminants and adverse effects of climate change*’. The biomonitoring programme has followed more than 25 years of environmental contaminants in air, sediment and biota in the Arctic.

POPs are organic chemicals that are persistent in the environment; they have the potential to be transported over long distances and bioaccumulated in wildlife and humans in the Arctic (Macdonald et al. 2000). The Stockholm convention on POPs initially listed 12 substances for elimination or control under its Annexes and currently there are 23 POPs listed in the Stockholm conventions. Although use of POPs has been either phased out or limited, POPs still exist in humans and biota. Levels and existence of POPs vary considerably between geographical areas and between species.

Trend data are used to assess the stability of contaminants in ecosystems and provide a first warning when potentially harmful contaminants may be elevating in the ecosystem. In addition, trend data are also valuable to examine the impact of regulations and regulator policy to limit the input of environmental contaminants to the environment (Rigét et al. 2010).

**Fig. 1:**
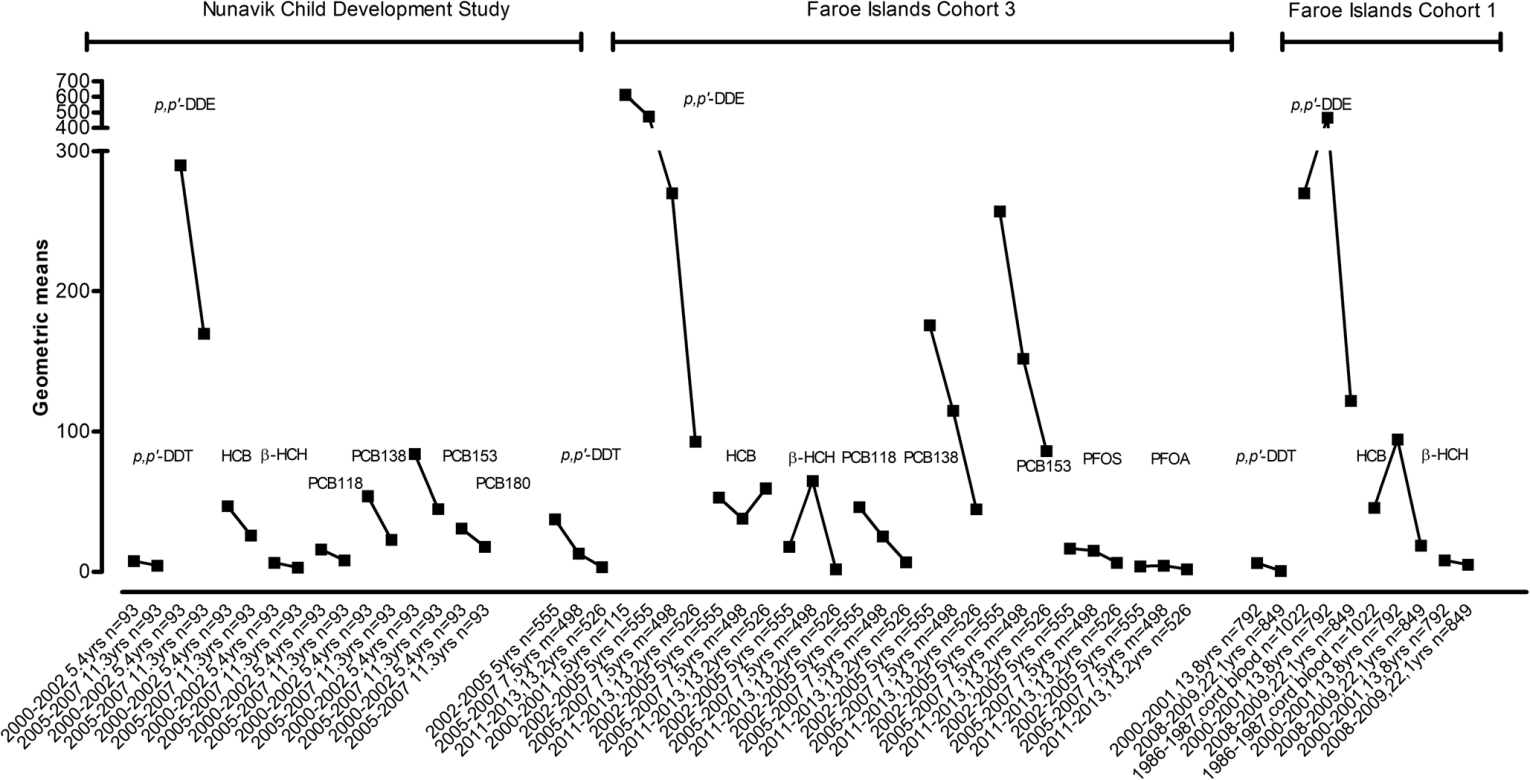
Trends of blood POP concentrations in children from the Nunavik Child Development Study, Canada and Faroe Islands Cohort 3. Data presented as geometric means. POPs and OCs are in μg/kg plasma lipid. PFCs are in μg/L

**Fig. 2:**
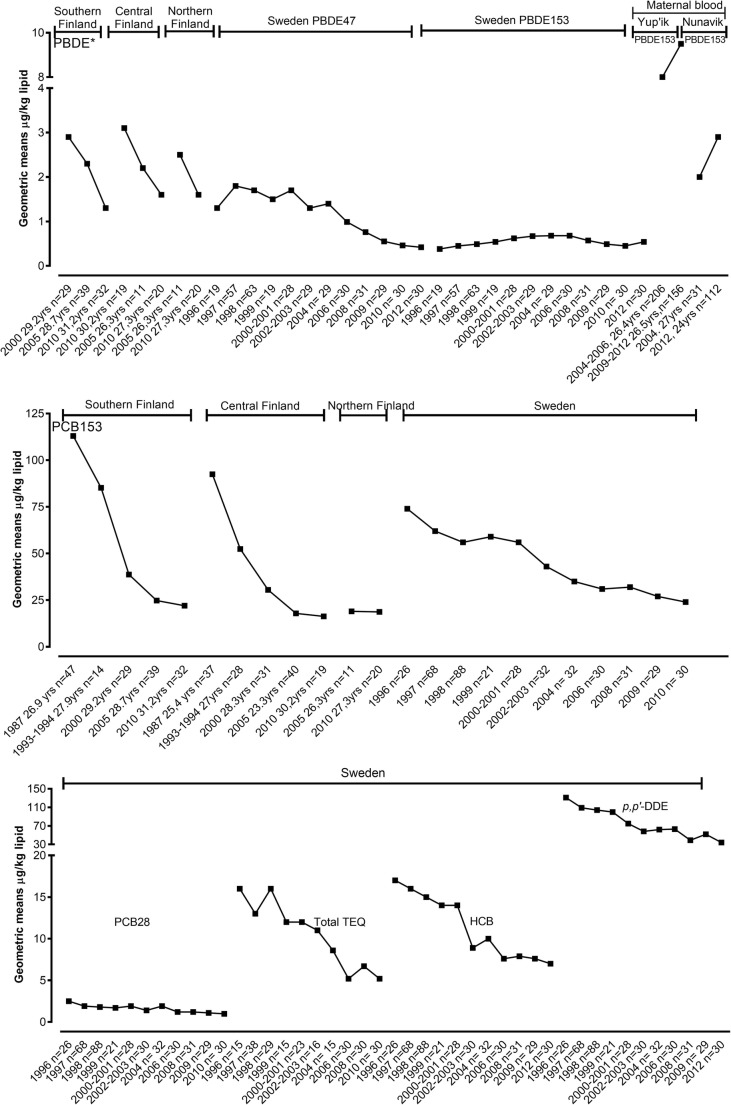
Trends of POP geometric means in breast milk samples (μg/kg lipid) from Finnish and Swedish first-time mothers. Breast milk was collected 3 weeks after delivery. Data are represented for the specific period of sampling. PDBEs also include data from Yup’ik and Nunavik maternal blood (μg/kg plasma lipid). PBDE in Finnish breast milk represented PBDE47 + PBDE99 + PBDE100 + PBDE153 + PBDE209

**Fig. 3:**
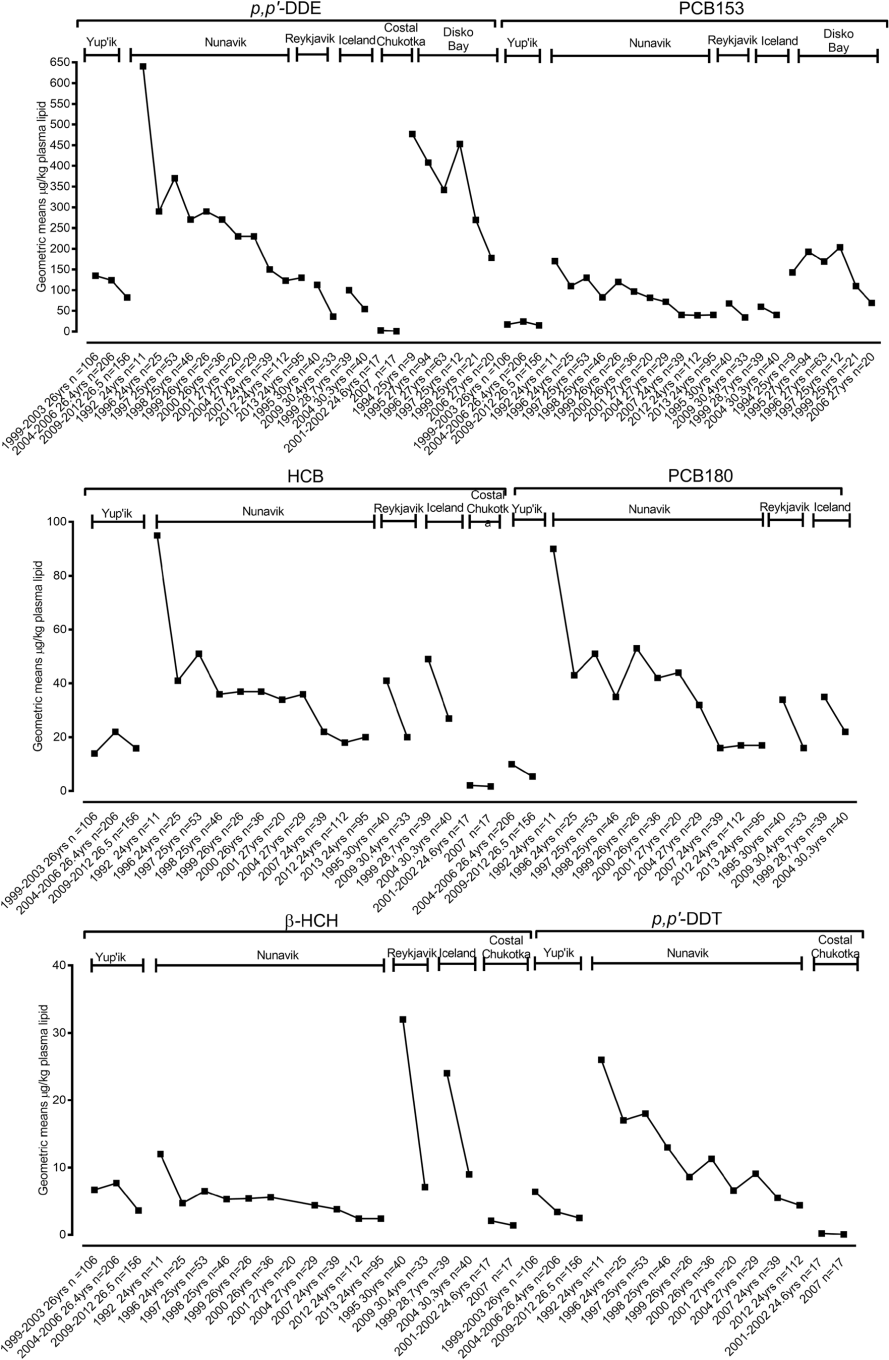

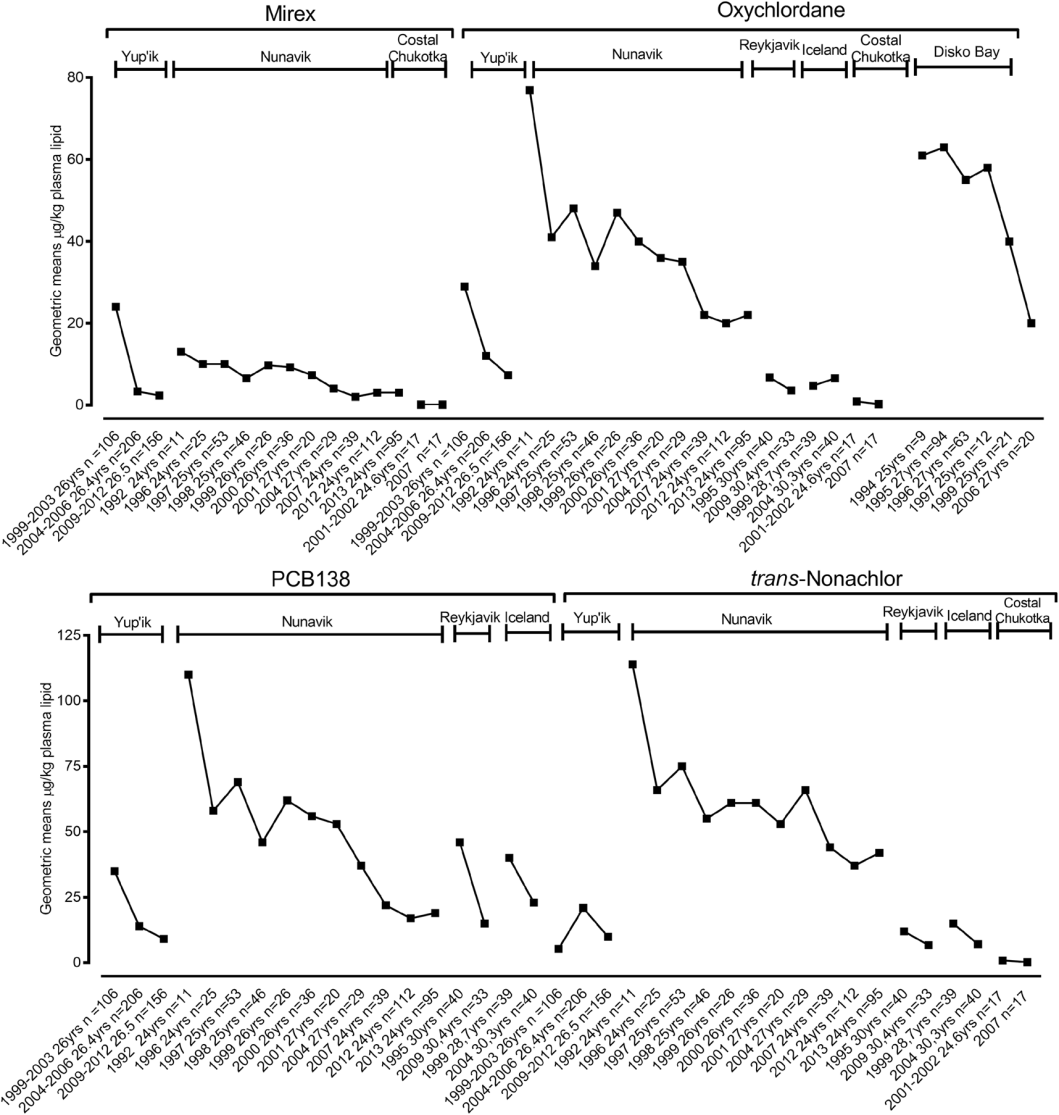
Trends of POPs in maternal blood. Data represented as geometric means (μg/kg plasma lipid) for the specific location and period of time

**Fig. 4:**
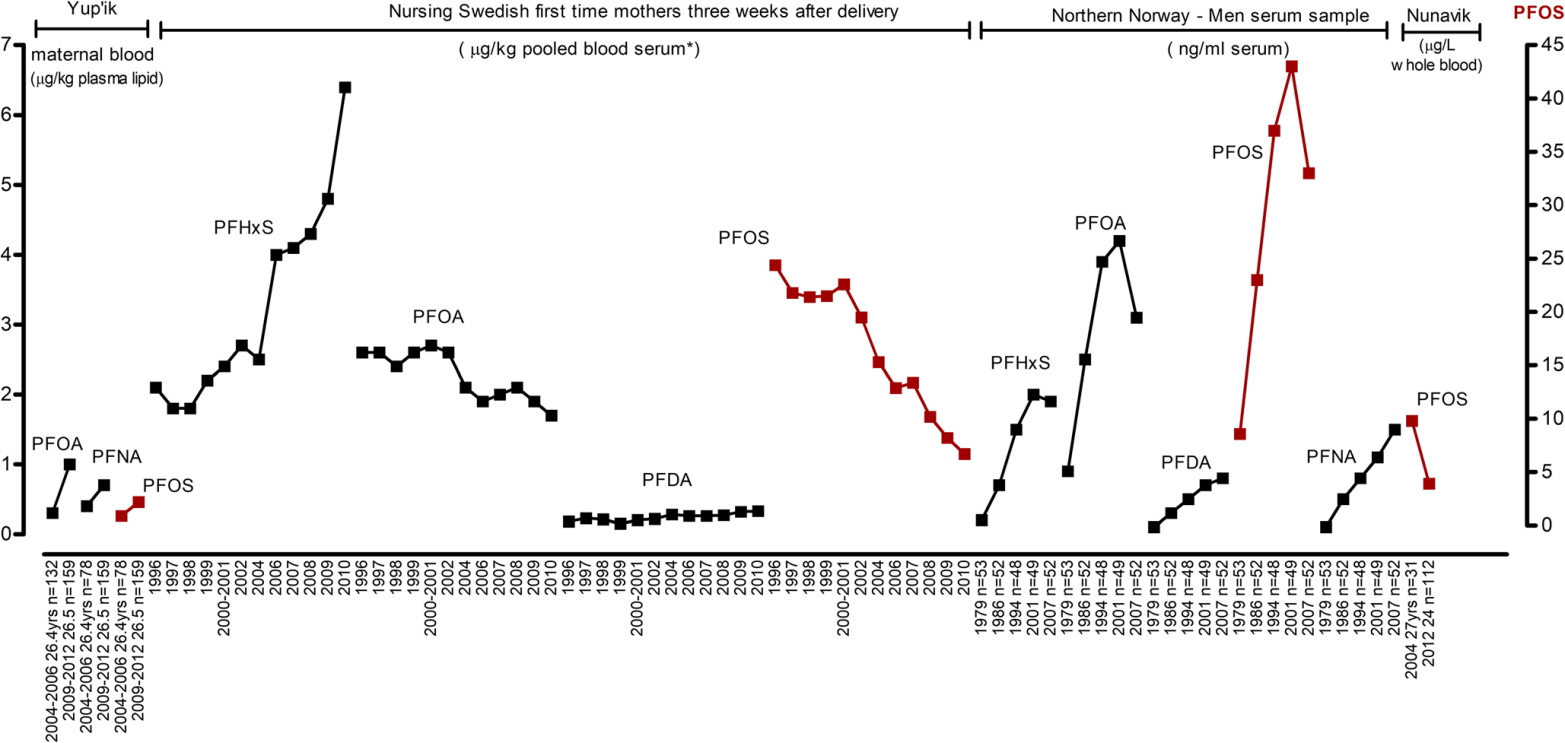
Trends of PFC geometric means (μg/kg plasma lipid) in Yup’ik maternal blood (μg/kg pooled blood serum) drawn 3 weeks after delivery from nursing Swedish first-time mothers and (μg/L whole blood) in Nunavik maternal blood, and median (ng/ml serum) in men serum sample from Northern Norway. Data represented for the specific period of sampling. *Three pools per year were analysed, with serum from 2 to 25 individuals in each pool

Arctic environment and ecosystem changes are expected to impact directly or indirectly the distribution profiles of environmental contaminants in the Arctic (Kallenborn et al. 2012; Macdonald et al. 2005). The focus area of AMAP reports was to monitor levels of environmental contaminants and to assess the health effects connected with detected levels in the Arctic. It is important to use comparable data (like study design and analysis methods of contaminants) over the time periods monitored. Therefore, this review examines data that have been reported in various AMAP reports. Data presented by AMAP depend on each of the Arctic countries’ National Implementation Plans in order to produce relevant information needed for Arctic monitoring research. AMAP monitoring data rely on the Ring Test to ensure that contaminants data from all participating laboratories are of high quality and comparability (Adlard et al. 2018). In addition, this review examines data that have been reported in the Russian scientific literature to give insight into the necessity to monitor contaminants and the approaches to health intervention in Russian Federation. The largest Russian domestic database entitled the Scientific Electronic Library ‘eLibrary.ru’ was used to identify publications relevant to this review among the Russian research literature. The types of the publication to conduct the search were inclusive to the next types: research journal articles, books and academic dissertations. The period of search was not limited to specific years and resulted to include papers from 2006 to 2017. A search was conducted in March 2018 using the following terms: contaminants, Arctic pollution, persistent toxic substances (and their individual names), human exposure to contaminants, levels of exposure, harmful effects on health etc. The aim of this review is to provide a firm basis for future levels and effects of pollutants in humans of the Arctic under climate and environmental changes. Principal findings are illustrated in terms of temporal trends of POPs and metals (Figs. 1, 2, 3 and 4), a summary of percentage changes of contaminants for a specific location and period of time, and health outcomes associated with contaminants based on data published on AMAP assessment 2015 (AMAP 2015).

## Temporal trends of contaminants in humans of the Arctic

### Trends of POPs in children

There are only a few trend studies measured from the same children. POP concentrations among children from the Nunavik child development study and Faroe Islands Cohort 3 are decreasing as shown in Fig. 1.

In the Nunavik child development study, there were two time points from the same children at average ages of 5 and 11 years. The reduction in POP concentration could be due to reduction in either environmental exposure or time intervals after breast feeding. Or, more likely, the changes could be due to the increase of body mass by age and the dilution of lipophilic contaminants. On the other hand, Faroe Islands Cohort 3 provided three time points of biomonitoring for all contaminants except for *p*,*p*′-DDE, in which four time points were monitored. Biomonitoring data showed a downward trend in levels of POPs in this cohort, excluding HCB and β-HCH. HCB concentrations decreased between 5 years and 7.5 years old, followed by an increase at the age of 13.2 years. HCB fluctuation shows no trend and its concentration remained relatively stable from 2002 to 2005 (53.2 μg/kg plasma lipid) to 2011–2012 (59.5 μg/kg plasma lipid). β-HCH shows an elevation at the age of 7.5 years and then sharp reduction by the age of 13 years (Heilmann et al. 2010; Tang-Péronard et al. 2014). In general, biomonitoring data showed a decline in levels of contaminants in the Faroe Islands Cohort 1, even though there was an increase in *p*,*p*′-DDE and β-HCH levels between birth and 13 years, before concentrations decreased when cohort participants were 22.1 years old.

Changes in ∑PCB [(PCB138 + PCB153 + PCB180) × 2] concentrations from the Faroe Islands Cohort 3 participants show a steady reduction of concentrations from children at age 1.5 to 13.2 years (1171, 1130, 750 and 347 μg/kg plasma lipid at age 1.5, 5.0, 7.5 and 13.2 years, respectively). ∑PCB trends from the Faroe Islands Cohort 1 participants demonstrate a decline from cord blood to the sampling ending at age of 22 years (604 and 443 μg/kg plasma lipid, respectively). Although ∑PCB showed a sharp increase from cord blood to 6.9 years old (604 and 1525 μg/kg plasma lipid, respectively), the follow-up years of biomonitoring showed a declining trend in levels of ∑PCB (AMAP 2015).

### Trends of POPs in breast milk

Trends of POPs geometric means in breast milk from Swedish and Finnish mothers are presented in Fig. 2. Breast milk samples from first-time Swedish mothers were collected 3 weeks after delivery over a time series between 1996 and 2012. Data showed a constant decline in trends for PCB28, PCB153, total TEQ, HCB, *p*,*p*′-DDE and PBDE47 from Swedish mothers, with the exception of PBDE153. PBDE153 exhibited fluctuated patterns, with the highest concentration detected in 2004–2006 and a lightly elevating trend during the study period. More biomonitoring data points are needed to investigate the unclear pattern of PBDE153 (Lignell et al. 2014). POP levels were measured in Finnish mothers from three different locations including Northern Finland. The length of time series covered differs between contaminants and locations. However, ∑PBDE levels decreased as well as PCB153 in all three Finnish locations, even though PCB153 remained stable from 19.0 to 18.7 μg/kg plasma lipid, during 2005 and 2010, respectively.

### Trends of POPs in maternal blood

Trends of POPs in maternal blood for the specific location and period are shown in Fig. 3. Trends of oxychlordane, *p*,*p*′-DDT, *p*,*p*′-DDE, Mirex and PCB138 in maternal blood from Yup’ik, Alaska declined steadily between 1999 and 2003 and 2009–2012. PCB180 also declined between 2004 and 2006 and 2009–2012. Levels of trans-nonachlor and HCB declined between 2004 and 2006 and 2009–2012, but the levels are still higher than the starting biomonitoring points during 1999–2003. PCB153 and β-HCH declined between 2004 and 2006 and 2009–2012, and the 2009–2012 levels are lower than the starting biomonitoring points during 1999–2003.

The POP concentrations in pregnant Inuit women from Nunavik, Canada decreased significantly over time. These included oxychlordane, trans-nonachlor, *p*,*p*′-DDT, *p*,*p*′-DDE, HCB, β-HCH, Mirex, PCB138, PCB180 and PCB153.

Despite limited data points, data showed an overall decrease in blood concentration of certain POPs in pregnant women from all Iceland as well as from Nunavik in their third trimester. Oxychlordane increased between 1999 and 2004 in Icelandic maternal blood, but it decreased between 1995 and 2009 in Reykjavik.

Data from maternal blood samples from the coastal Chukotka 2001–2003 birth cohort showed a reduction in trends in oxychlordane, *p*,*p*′-DDT, *p*,*p*′-DDE, HCB, β-HCH, Mirex and ∑PCBs between 2001 and 2002 and 2007 (Dudarev et al. 2010). Likewise, blood samples from pregnant Inuit women from Disk Bay, Greenland showed a declining trend for oxychlordane, *p*,*p*′-DDE and PCB153 between 1994 and 2006 (Deutch and Hansen 2000; Deutch et al. 2007; Kruger et al. 2008).

### Trends of PFCs

Trends of PFC from different cohorts and different human biological matrices are collected in Fig. 4. The concentration of PFCs in Yup’ik maternal blood showed increasing trends for PFOA, PFNA and PFOS over the time period from 2004 to 2006 to 2009–2012, while the level of PFDA remained similar. The increasing trend was also reported from maternal blood collected 3 weeks after delivery from nursing Swedish first-time mothers for PFHxS and PFDA, while PFOS and PFOA decreased over the time period from 1996 to 2009 (Glynn et al. 2011, 2012). PFOS declined in Nunavik maternal blood during the 2004 to 2007 sampling period.

Figure 4 also shows trends of PFCs in serum samples from Northern Norway men (Nøst et al. 2014). The Tromsø study showed that PFHxS, PFOA and PFOS concentrations increased from 1979 to 2001 and decreased from 2001 to 2007. In addition, the Tromsø study showed significantly declining trends for several POPs in the same Norwegian men between 1979 and 2007 (Supplementary Fig. S1; Nøst et al. 2013). On the other hand, the concentrations of PFNA and PFDA showed increasing trends from 1979 to 2007.

### Trends of metals

**Fig. 5:**
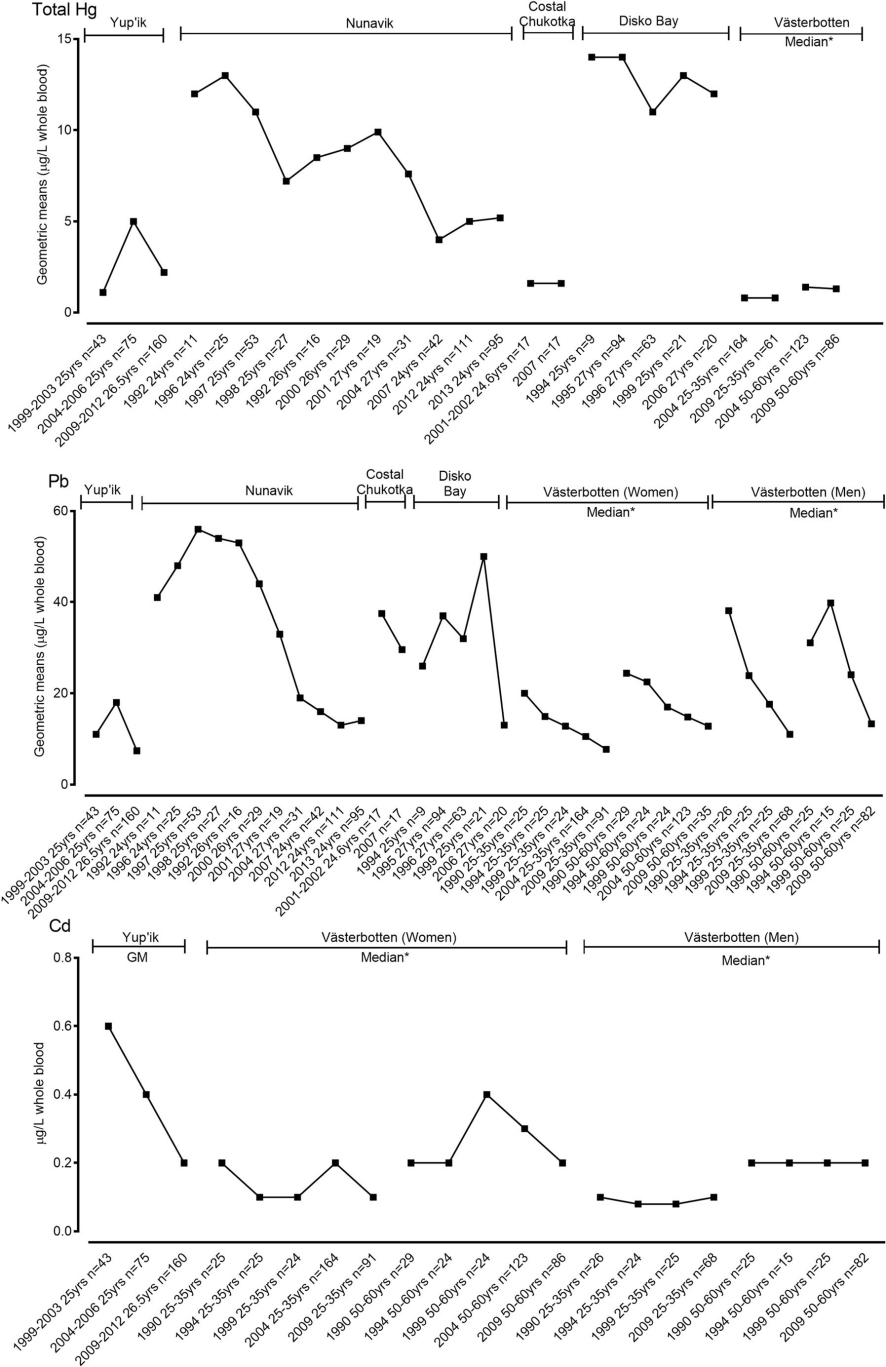
Trends of total Hg, Pb and Cd geometric means (μg/L whole blood) in Yup’ik, Nunavik, coastal Chukotka and Disko Bay maternal blood and *median (μg/L whole blood) in women and men from Västerbotten, Sweden. Data represented for the specific period of sampling

Trends of total Hg, Pb and Cd geometric means (μg/L whole blood) in Yup’ik, Nunavik, coastal Chukotka and Disko Bay maternal blood and median (mg/L whole blood) in women and men from Västerbotten, Sweden are shown in Fig. 5.

Trends of total Hg, Pb and Cd: The level of Cd decreased steadily in Yup’ik maternal blood between 1999 and 2003 and 2009–2012. Levels of Hg and Pb decreased from 2009 to 2012 by almost half compared to the values detected from 2004 to 2009. However, the level of Hg during 2009–2012 (2.2 μg/L whole blood) was still higher than the level detected at the beginning of the biomonitoring from 1999 to 2003 (1.1 μg/L whole blood).

Trends of metal concentrations in the blood of pregnant women from Nunavik, Canada showed significant declines in Pb and total Hg among Inuit in Nunavik between 1992 and 2013. In coastal Chukotka maternal blood, the concentration of total Hg was stable (1.6 μg/L whole blood), while Pb concentration decreased between 2001 and 2002 and 2007 with data available only from two points. Despite the fluctuation of both total Hg and Pb concentrations in pregnant Inuit women from Disko Bay, Greenland (Deutch and Hansen 2000; Deutch et al. 2007; Kruger et al. 2008), both total Hg and Pb continued to decline over the biomonitoring period.

The concentration of THg levels in blood from women from Västerbotten, Sweden showed a stable trend. Women 25–35 years of age had lower levels of THg than women 50–60 years of age. On the other hand, there is a declining trend of blood Pb concentration for both men and women from both age groups (25–35 and 20–60 years old). The trends of Cd concentrations (μg/L whole blood) in men and women between 1999 and 2009 for both age groups (25–35 and 20–60 years old) were stable despite a minor fluctuation in Cd levels (Sundkvist et al. 2011).

## Summary of percentage changes in contaminants in humans of the Arctic

Humans are exposed to environmental contaminants through ingestion, inhalation and dermal absorption. Exposure to contaminants depends on the contaminant concentration, and frequency and duration of contact. While human exposure through traditional diet is one of the main sources of human exposure in the Arctic, the level of contaminants in human biological matrices provides the aggregate exposure from different routes.

Supplementary Table S1 shows a summary of percentage changes for contaminants in human biological matrices for a specific location and period of sampling. While the percentage change of a particular environmental contaminant is vital to evaluate its input trend in the ecosystem, it does not illustrate the level of exposure as well as the health consequences. Therefore, detailed trend data are valuable. Figures 1, 2, 3, 4 and 5 illustrate trends of POPs in blood of children, in breast milk, in maternal blood and trends of PFCs in human blood and serum as well as trends of total Hg, Pb and Cd in humans for a specific location and period of sampling.

The data presented were collected over a varied period for several locations in the Arctic. Contaminant levels are declining in most of the monitored Arctic locations. Contrary to other monitored populations, oxychlordane is increasing only in Icelandic maternal blood in the third trimester between 1999 and 2004, even though oxychlordane levels in 2009 declined markedly from its concentrations measured in 1995 in Reykjavik. Hexachlorobenzene (HCB) levels in blood from the Faroe Islands Cohort 1 shows elevating trend between birth and 13 years of age, before its levels in blood decreased by age of 22 years. On the other hand, the Faroe Islands Cohort 3 biomonitoring data show declining trends in concentrations of contaminants in this particular cohort, with the exception of HCB. HCB shows a relatively stable trend from 2002 to 2005 (53.2 μg/kg plasma lipid) to 2011–2012 (59.5 μg/kg plasma lipid).

Certain contaminants such as PBDE153 ‘2,2′,4,4′,5,5′-hexabromodiphenyl ether’ increased in breast milk from first-time mothers in Sweden during the period of sampling from 1996 to 2012. The concentration of PBDE153 was higher in 2012 (0.54 μg/kg lipid) than in 1996 (0.38 μg/kg lipid). PBDE153 is still increasing in Yup’ik maternal blood. The levels of PBDE153 were greater in 2009–2012 than in 2004–2006, even though the other polybrominated PBDEs were lower in 2004–2006. There are limited data for PBDE153 in Nunavik pregnant Inuit blood. All POPs showed declining trends between 2004 and 2012. The exception is PBDE153, which elevated between 2004 and 2012.

The percentage of change in perfluorinated compounds ‘PFCs’ concentrations (μg/kg pooled serum) in blood drawn 3 weeks after delivery from nursing Swedish first-time mothers shows a general declining trend for both perfluorooctanesulphonic acid (PFOS) and perfluorooctanoic acid (PFOA), while perfluorohexanesulphonic acid (PFHxS) and perfluorodecanoic acid (PFDA) both show an increase over time between 1996 and 2010. In men from northern Norway, the change in the levels of PFCs analysed in serum samples showed discrepancies between the trends. The concentrations of PFOS and PFOA elevated 5-fold from 1979 to 2001 and declined by 26 and 23%, respectively, from 2001 to 2007. The levels of PFOS and PFOA hiked during 1994–2001 and 2001, respectively, while PFHxS elevated to 2001, but did not demonstrate a downward trend between 2001 and 2007. PFNA and PFDA illustrated upward trends during the study period (1979–2007). The level of PFOS was greater than other PFCs. The elevated levels of PFOS, PFNA and PFDA were correlated with marine mammals and fish consumption, while high concentration of PFOA was correlated with beef consumption. Increased levels of PFHxS and PFNA were significantly correlated with the consumption of game. The trend of the concentrations of PFCs in Yup’ik maternal blood from Alaska illustrated that several PFCs concentrations were higher 2009–2012 than in 2004–2006. PFOS and PFOA levels were increasing in mothers over time. PFNA also showed a slight increase, while PFDA remained similar.

There has been a gradual decrease in the concentration of contaminants, with the exception of HCB, PBDE153 and PFCs, measured in the Arctic and the reduction varied between different populations. The geographical differences in the concentrations and trends of several contaminants reflect the distinct cultures, traditional lifestyles and dietary habits. The availability of commercial food has reduced the consumption of traditional foods in the Arctic (Curren et al. 2015). The elevated levels of PFOS, PFNA and PFDA were correlated with the consumption of marine mammals and fish, while a high concentration of PFOA was correlated with beef consumption. Increased levels of PFHxS and PFNA were significantly correlated with the consumption of game. High POP concentrations have been accumulating in lipid tissues of Arctic marine mammals over the last several decades. Key findings of the comprehensive Arctic research project ArcRisk (https://cordis.europa.eu/result/rcn/161281_en.html) showed that species of marine mammals contain a variety of legacy POPs that have been included in the Stockholm Convention for over a decade as well as PFAS (Carlsson et al. 2018). The highest mean concentrations of most POPs, metals and PFCs are connected to high consumption of marine mammals, game and fish (Berg et al. 2014; Health Canada 2010, 2013; Weihe and Joensen 2012).

Legacy POPs and their production and usage are globally regulated under the Stockholm Convention, which entered into force in 2004. As of 2018, in addition to the 12 initial POPs, 16 new chemicals have been added (http://chm.pops.int/TheConvention/ThePOPs/TheNewPOPs/tabid/2511/Default.aspx). Biomonitoring data showed declining trends of all POPs listed under the Stockholm Convention (Annex A ‘Elimination’) except hexachlorobenzene (HCB) and hexabromodiphenyl ether (PBDE-153). HCB shows fluctuation with relatively stable concentrations in blood levels of children from Faroe Islands Cohort 3, while it shows declining trends in maternal blood between 2004 and 2006 and 2009–2012 from Yup’ik. However, its levels are still higher than the starting points in 1999–2003. On the other hand, elevated patterns of PBDE153 in maternal blood from Yup’ik and Nunavik were reported between 2004 and 2012 based on two time points. The biomonitoring data of HCB and PBDE153 are scarce and available for a limited time range, and it may take several years before time series are available that are suitable for reliable trend assessment.

Legacy POPs in the Arctic have shown declining trends in human biological matrices in the Arctic region due to strict international regulations. On the other hand, new chemicals have been detected in Arctic human populations. Some of those meet the POPs criteria. Elevated perfluorohexane sulfonic acid (PFHxS) levels were identified in serum samples of nursing Swedish first-time mothers and men in Northern Norway. PFHxS is still under review by the POPs Review Committee. As shown with other listed POPs, PFHxS could show a deceasing trend after the international regulation of production and usage. Legislation and long-term monitoring are vital for limiting the levels and health-related risks/effects from exposure to environmental contaminants. On the other hand, PFOS, which is among the 16 new POPs under the Stockholm Convention, showed a decreasing trend but is still higher than the starting biomonitoring point of Northern Norway men. Its trend is steadily increasing levels in the serum samples of nursing Swedish first-time mothers. PFOA, PFNA and PFOS showed increasing trends in blood samples of Yup’ik mothers, but data are only available for two time points. Despite the phase-out of listed POPs production and usage, contaminant circulation in the environment through their environmental fate will remain. Environmental factors and the impact of climate change on temperature will affect the volatilisation and distribution of POPs. For instance, despite the general declining trend of PCBs levels in human biological matrices and biota, modelling of the atmospheric PCB composition and behaviour showed some increase in environmental concentrations in a warmer climate (Carlsson et al. 2018).

## Health outcomes associated with contaminant levels in humans of the Arctic

Several cohorts’ studies were established in the circumpolar area to examine the association between exposure to contaminants and health outcomes. Parental exposure to MeHg has been linked to neurobehavioural effects, up to the age of 22, such as decreased motor function, attention span, verbal ability, memory and defects in general mental ability in the Faroe Islands cohort studies (Debes et al. 2006, 2016; Grandjean et al. 2004; Murata et al. 2004). In addition, the Nunavik cohort study reported that children at age of 11 years developed poorer early processing of visual information, lower estimated IQ, poorer comprehension and perceptual reasoning, poorer memory functions, and increased risk of attention problems and ADHD behaviour due to parental exposure to Hg (Boucher et al. 2010; Geier et al. 2012). Therefore, parental and infant exposure to mercury increases the risk of neurobehavioural defects during child development.

In addition, Hg was found to be correlated with adverse cardiovascular effects in exposed cohorts. High Hg concentration in cord blood was reported to be associated with decreased heart rate variability in children at ages 7 and 14 years from the Faroe Islands. In children from Nunavik, child blood Hg levels were correlated with reduction of overall heart rate variability parameters. Hg adversely impacts the cardiovascular system not only in children but also in adults. Exposure to Hg was associated with elevated blood pressure among adults from the Faroe Islands and Nunavik, and decreased heart rate variability in adults from Nunavik (Grandjean et al. 2004; Valera et al. 2008).

Organochlorines, especially dioxin-like PCB congeners, affect the immune system development (Jusko et al. 2010; Ten Tusscher et al. 2003). Different cohort studies in Nunavik reported that parental exposure to organochlorines increases the susceptibility to infectious diseases, particularly otitis media among Inuit children (Dallaire et al. 2004, 2006; Dewailly et al. 2000). In addition to organochlorines, perfluorinated compounds may inhibit immune function. Results from the Faroese cohort studies showed that perfluorinated compounds strongly, negatively affected serum antibody concentrations more than organochlorine compounds during developmental and perinatal exposure (Grandjean et al. 2012a; Heilmann et al. 2006, 2010).

Dioxin-like PCBs were reported to impact bone quality. PCB105 and PCB118 were inversely associated with the bone stiffness index in Cree women of Eastern James Bay (Canada). On the other hand, a lack of correlation between dioxin-like PCBs and stiffness index was reported in Inuit women from Nunavik (Paunescu et al. 2013a, 2013b).

Several epidemiological case–control studies reported POP as a major risk factor for developing diabetes and metabolic syndrome (Carpenter 2008; Everett and Matheson 2010; Lee et al. 2007; Patel et al. 2010). These findings suggest that POP may initiate gene–environment interactions with impacts on insulin resistance and/or secretion (Weihe et al. 2016).

PCB153 in blood was not correlated with the proportion of morphologically normal sperm, but it was inversely related to sperm motility in a semen quality control study conducted in Greenland during 2004 (Dufour 1988). Moreover, in Faroese young men, high PCB levels were associated with low semen quality (Bonefeld-Jorgensen 2010; Bonefeld-Jørgensen et al. 2014). Also, prenatal and postnatal exposure to high levels of PCBs induced serum steroid hormone-binding globulin in Faroese men (Grandjean et al. 2012b). In addition to PCBs, perfluorinated compounds were reported as reproductive system function disruptors. Exposure to perfluorinated compounds and human semen quality was monitored in the Arctic study (CLEAR); it was reported that exposure to perfluorinated compounds was not correlated to male reproductive functions, including reproductive hormones and markers of sperm DNA damage. On the other hand, PFOS was associated with more abnormal sperm morphology (Specht et al. 2012; Toft et al. 2012). In addition, high levels of PFCs in blood were adversely associated with longer menstrual cycles in women (Lyngsø et al. 2014).

Several epidemiological studies in the Arctic reported the endocrine-disruptive potential of POPs. Prenatal exposure to high levels of PCBs was associated with lower serum luteinising hormone and testosterone in Faroe Islands boys. Lower serum luteinising hormone leads to delayed puberty, and it might be due to the interactions of non-dioxin-like PCBs with the central hypothalamo-pituitary mechanism (Grandjean et al. 2012b). In addition, several studies have evaluated the association between thyroid hormone disruption and exposure to environmental contaminants at different life stages. Studies found that perinatal exposure to PCBs reduces thyroid hormone in the offspring. Exposure to PCBs interferes with thyroid hormone homeostasis in adults, while a significant correlation between POPs and thyroid hormones was also reported in ageing residents of upper Hudson River communities in the USA (Bloom et al. 2014; Boas et al. 2012; Salay and Garabrant 2009). Several polyhalogenated compounds have been associated with thyroid hormone parameter modulations in Inuit adults from Nunavik (Dallaire et al. 2008, 2009a, 2009b).

The International Agency for Research on cancer classified PCBs and PBDES as human carcinogens and possible human carcinogen compounds, respectively. Generally, the carcinogenicity potential of PCBs is attributed to the induction of reactive oxygen species, genotoxicity effects, immune suppression and endocrine effects. The carcinogenicity potential of dioxin-like PCBs is initiated mainly via the AhR activation mechanism (Lauby-Secretan et al. 2013; Pestana et al. 2015). Several environmental contaminants such as perfluorinated compounds, arsenic, cadmium, lead, mercury, chromium, cobalt and nickel are suspected carcinogens that act through an oxidative stress mechanism (Eriksen et al. 2010; Filipic 2012; Hartwig 2013; Hu and Hu 2009; O'Brien et al. 2005; Schwerdtle et al. 2010; Wielsøe et al. 2015). Epidemiological studies reported associations between exposure and cancer risk. In southern Quebec, increased levels of PCB105, PCB118 and PCB156 were associated with breast cancer (Demers et al. 2002). In Greenlandic Inuit, serum PFC levels were associated significantly with breast cancer risk, and the breast cancer cases had a significantly higher concentration of PCBs at the highest quartiles (Bonefeld-Jorgensen et al. 2011). In Icelandic Inuit women, the BRCA1 founder mutation and polymorphisms in CYP1A1 and CYP17 elevate the risk of breast cancer, and that risk increases with higher serum levels of PFOS and PFOA (Ghisari et al. 2013).

## Contaminant monitoring and health implications discussed in the Russian scientific literature

### Monitoring needs

The Arctic regions of the Russian Federation cover around half of the Arctic and two thirds of the population of the Arctic is living there (Emelyanova 2017), and there is only limited trend data of contaminants. We focused on data published in Russian peer-reviewed scientific journals to give insight into the necessity to monitor contaminants and the approach to health intervention in the Russian Federation. The assessment of the role of environmental factors including contaminants in affecting the health of Russian Arctic inhabitants is a topical issue in the Russian scientific literature. Obtaining such assessments in practice is associated with considerable difficulties and the need for a combination of approaches from various disciplines (medicine, environmental epidemiology, etc.) (Zakharov et al. 2018). There is academic agreement that the existing programmes are insufficiently effective, and also that there is an urgent demand to enhance country approaches to biomonitoring for proper assessment, forecast and administration of health risks related to biotransportation of persistent toxic substances (PTS) over a long distance into the Arctic food chains and ecosystems (Chashchin et al. 2012, 2017; Dudarev and Odland 2017; Khurtsilava et al. 2017; Meltser et al. 2016).

### Measurement of effect

Several international research exercise have been carried out in recent decades in the Russian Arctic with the aim to analyse the impact of toxic environmental contaminants on population health under the aegis of AMAP (Chashchin et al. 2012; Dudarev and Chupakhin 2012; Dudarev et al. 2012, 2017; Dudarev and Odland 2017; Khurtsilava et al. 2017; Konoplev et al. 2006; Konoplev and Tsaturov 2014).

#### Chukotka and Nenets autonomous areas

In territorial terms, adverse effects on human health due to exposure to harmful substances found in the Russian northern territories have been largely focused on the Chukotka and Nenets autonomous areas (a.a.). There were several waves of data collection in the form of cohort epidemiological studies among Indigenous peoples in Chukotka (coastal and inland) and Nenets a.a. from 2000 to 2010. These collections, in the form of blood, food and household item sampling, allowed the evaluation of concentrations of persistent pollutants that may cause profound health concerns.

The AMAP survey in 2001 (AMAP 2004) monitored indigenous cohorts and measured blood concentrations of persistent contaminants including 14 PCB congeners and 13 organochlorine pesticides and metals (wave 2001–2002). The indigenous people from Chukotka were registered to have significantly greater levels of PCBs, hexachlorobenzene, chlordane derivatives alpha-chlordane, beta-hexachlorocyclohexane and lead than found among population residing in the Nenets a.a. There, native people from the coastal areas showed a lower percent of low chlorinated and dioxin-like PCB congeners (compared to native people from the mainland) with a significant share of this ‘triad’ in the number of PCBs.

After 10 years of observation (wave 2009–2010), a noticeable reduction in the mean serum concentrations of DDT and 4,4-DDE, 55 and 51% reduction, respectively, was observed (Chashchin et al. 2012). The found variations for the studied cohorts may be due to regional dietary patterns or different contaminant elimination patterns in the Arctic. Indigenous people of coastal Chukotka have much higher DDT/DDE blood levels than in the inland inhabitants of Chukotka and the Nenets area by cause of consuming more fish and marine mammals most exposed to global DDT pollution.

Special attention was paid to reproductive health, and for this a population group consisting of mothers and their unborn children was selected in Chukotka. In its coastal part, the ratio of the analysed PCB congeners in the blood samples remained unchanged 5 years after the first survey. The composition of PCB congeners in the blood of the continental population significantly differs from those in the food, while in coastal residents the composition was similar to the composition of PCBs found in samples of marine mammals in that area. The content of PCB congeners in the local producers of pollution has no resemblance to the composition of PCBs in the blood of local population. The blood concentrations of 4.4-DDE in the women of reproductive age of coastal Chukotka are similar to findings in other Russian Arctic regions, marginally lower than in Greenland, but substantially higher than in Alaska, Canada and Scandinavian countries (Dudarev and Chupakhin 2012). Also, the Chukotka blood samples identified no associations of elevated levels of POPs and metals with premature births and low birth weight; a reverse but not statistically significant relationship was found with regard to POPs. Mothers’ blood concentrations of POPs were higher in the case of stillbirths and congenital malformations. Higher POP blood concentrations were noticed in females with earlier menarche, compressed menstrual cycle and longer menstrual bleeding, however not significantly. Females that were more exposed to PCBs and other POPs gave birth to girls more often. With regard to any PTS and the dose range, there was no elevated relative risk of harmful pregnancy outcomes established (Dudarev et al. 2010a; Dudarev and Chupakhin 2014; Dudarev and Chashchin 2006).

During the years 2003–2004 and later, some mitigation actions were attempted based on recommendations by the international AMAP expert panel. These actions addressed the disposition and detoxing activities on local sources of persistent contaminants and reducing the risk of related harmful impact on health. However, the results confirm the ineffectiveness of implemented actions for the mitigation of population health effects in both the Chukotka and Nenets areas. For example, Khurtsilava et al. found the average yearly incidence of diseases stemming from the harmful impact of PTS, in particular, neoplasms, endocrine system disorders, immunodeficiency and congenital malformations showed a clear increasing trend during the decade of observation (Khurtsilava et al. 2017). This is in contrast to majority of the other groups of diseases in the population. It was concluded that the duration of re-calculating PTS blood concentrations was not proper due to the longer half-life of many studied PTS. The health influence from of global cross-border transportation of PTS must also be underestimated, as a result of the observed high contamination of commercial species of birds, fish and coastal aquatic animals that create a profound share of the local diet of the Arctic indigenous peoples (Khurtsilava et al. 2017).

#### Murmansk region (Kola peninsula)

Health risks caused by metals found in regional food items and drinking water were assessed in several areas of the Murmansk region subject to industrial emissions (Dudarev et al. 2015a, 2016). The authors analysed the levels of 13 metals in foods and 15 metals in water sources. Those are the metals with increased levels compared to permitted daily intake (i.e. overall, cadmium showed 22% higher intake, mercury 40%, nickel 66% and arsenic showed as high as 157% higher intake). The highest carcinogenic risks to population health come from the highly toxic nickel; hence, the authors recommended the reduction/exclusion of intake of some local foods (mainly fish and mushrooms) and enhancing drinkable water purification from nickel, or usage of other sources of clear water (Dudarev et al. 2015).

In addition, the values of aluminium element in the city of Kirovsk and for nickel in Zapolarny and Nikel cities significantly (two to five times) exceeded the maximum allowable concentrations (both in water sources and in drinkable waters). In all the studied cities, significantly increased concentrations of iron and other metals were seen during the phase of water transport from the source to the city supply, which indicates the need to modernise the water supply system and replace its old elements (Dudarev et al. 2015b).

The blood samples collected in the Pechenga area of the Murmansk region were additionally analysed for gender differences. Male serum samples had higher concentrations of lead, zinc, nickel and mercury. Female samples showed higher levels of manganese, cobalt, cuprum and arsenic than that in males. Pregnant women had the lowest concentrations of metals in their blood. Comparing the results to the WHO reference, levels of metals in whole blood demonstrated extremely high percentages of excess (and significant values) of manganese and nickel. Maximum manganese concentrations in blood reached 300 μg/L, and those of nickel, 100 μg/L. Average blood concentrations of mercury in the examined samples did not reach the most severe level of concern for all ages (5 μg/L), and shares of men and women with blood concentrations of mercury over this limit were relatively small. Average blood concentrations of lead in the examinees did not reach the lower allowable level (50 μg/L) (Dudarev et al. 2016).

A comparative assessment of the prevalence rate of diabetes mellitus (type 1 and 2 and risk factors) was done in Kola Lapland comparing Indigenous and non-Indigenous populations. Exposure levels to POPs and the association of the diabetes status with exposure levels in Indigenous people to POPs were evaluated. According to predisposition criteria for diabetes mellitus, the Indigenous population in remote rural settlements holds a minimum risk. Obesity, elevated blood glucose levels and the presence of diabetes itself are associated with higher POP concentrations in the blood of examined people, but statistically significant links were not revealed (Dudarev and Nikanov 2012; Dudarev and Odland 2017).

### Approaches to health intervention

According to the results of studies of PTS and POPs in the Russian northern areas, which were undertaken in both the European and Siberian parts of the Russian Arctic, basic patterns were systemised and optimal actions were formulated to decrease the Northern peoples’ exposure to harmful substances. A set of actions was suggested to minimise the circulation of PTS-containing items in the Arctic (at all stages such as detecting, collecting and eliminating those), neutralising the soils in the territory of settlements, creating solutions for safe water consumption, implementing efficient control over the secure usage of chemicals and the levels of PTS in raw foods, and establishing guidelines for better protected food purchase, safe storage and meal cooking (Dudarev et al. 2010b).

Several authors highlighted that practices establishing a causal link between the environmental hazardous substances and harmful health effects in Russia often do not follow the evidence-based approach. The issues of designing the study, data collection and technical analysis in the spirit of proper collection and evidence-based interpretation of environmental pollution, which may lead to harmful effects on health, have been topics of discussion among Russian scholars. It has been shown that related scientific models cannot be considered as evidence-based if the model is unable to provide a reliable prediction of a harmful effect when the risk environment is a combination of different pollutants and conditions. Therefore, there is a need for further development of approaches to investigate the aggregated effect of several pollutants on health (Gorbanev et al. 2017).

Several scientists underlined the geographical and political isolation of the Russian Arctic zone regions as having an impact on the planning, availability and quality of medical aid, and also the response to contamination. They identified overall priorities in the strategic planning of a public health programme in the Russian Federation Arctic zone. Healthcare planning should be focused on foreground diseases, which make the largest contribution in disability-adjusted life years calculated for the indigenous Arctic population (Chashchin and Plakhin 2015). According to them, greater attention should be given to preventive and compensatory measures. Financial compensation for health damage due to high air contamination in the cities of the Russian Arctic has been addressed. A multifaceted mechanism of compensation was suggested based on hedging contamination forecasts to associate with estimated health risks as well as identifying sources of air pollution to fund compensatory benefits (more in Matesheva 2017) (Matesheva 2017).

## Conclusion

Biomonitoring data showed the time trends of metals and POPs, except PFCs, are declining, which indicates the importance of global actions to reduce emissions of contaminants. On the other hand, broad ranges of new chemicals of emerging Arctic concern have been identified. Examples include per- and polyfluoroalkyl substances, brominated, chlorinated and organophosphate-based flame retardants, polychlorinated naphthalenes, hexachlorobutadiene, pentachlorophenol and pentachloroanisole, organotins, polycyclic aromatic hydrocarbons and current-use pesticides (AMAP 2017). It is essential that more data are collected on levels and temporal trends of chemicals of emerging Arctic concern in different environmental mediums as well as in human biological media over wider geographical areas.

Studies on the impact of pollutants on human health are challenging to undertake with many other confounding factors influencing health at the same time. There have been several EU projects to investigate the link between exposure to environmental contaminants and their risks for human health. Human health risk assessment from exposure to contaminants could be evaluated through several approaches, i.e. long-term retrospective epidemiological studies, modelling, systematic review and toxicological cutoff reference values. Epidemiological studies that are established in the circumpolar area to investigate the link between exposure to contaminants and health outcomes showed links between exposure to contaminants and neurobehavioural, reproductive, cardiovascular, endocrine and carcinogenic effects. However, future epidemiological studies for new emerging compounds in the Arctic populations should be standardised. A systematic review that pooled research findings regarding the association between PCB exposure levels and secondary sex ratio revealed several limitations (Nieminen et al. 2013): (1) a limited number of relevant epidemiological data from the Arctic populations; (2) high heterogeneity in analysing and reporting findings; (3) lack of standardised, repeated studies of the same phenomena, for instance the size of the study, differences in the used analytical methods and genetic differences between the studied populations; (4) the use of various statistical methods in different publications to measure the same outcomes; (5) variation in the standard and quality of reporting between publications in terms of detailed descriptive statistics of the variables under study, and the unavailability of standard errors for regression coefficients or the mean differences in some reports. Despite the wealth of literatures and overall evidence for the positive association between exposure to some organochlorines and type 2 diabetes, meta-analysis failed to establish sufficient causality between exposure and type 2 diabetes due to high variation across studies. Taylor et al. found out that only 43 studies were eligible in their meta-analysis out of 2752 identified publications (Taylor et al. 2013). Moreover, Tuomisto and coworkers (Tuomisto et al. 2016) found out that establishing the causality between exposure to POPs and type 2 diabetes incidence requires detailed information on the analysis of food consumption as well as the pharmacokinetics of the studied compounds.

Future research should focus on new emerging contaminants as well as establishing toxicological cutoff points to evaluate the health consequences for humans. Furthermore, new approaches need to be developed to estimate the magnitudes of health effects of exposed populations as well as determine the effects of mixtures.

## Electronic supplementary material

Table S1 Percentage changes for contaminant monitoring trends in human biological matrices for the specific location and period of sampling. (PDF 157 kb)

Figure S1 Trends of contaminants concentrations (ng/g lipid) in serum samples of men in the Tromsø study (Nøst et al. 2014). (PDF 768 kb)
