# Supplementary material for: Temporal trends of contaminants in Arctic human populations
Source: Environ Sci Pollut Res Int. 2018 Aug 25;25(29):28834–50. doi: 10.1007/s11356-018-2936-8 (PMC6592971; doi:10.1007/s11356-018-2936-8)
Supplement: Supplementary file 1 — Percentage changes for contaminant monitoring trends in human biological matrices for the specific location and period of sampling. (PDF 157 kb) [file 11356_2018_2936_MOESM1_ESM.pdf]

Table S1:

Percentage changes for contaminant monitoring trends in human biological matrices for the specific location and period of sampling

Cohort	Biological matrices	Biomonitoring Period	Overall changes %
HCB Hexachlorobenzene			
Sweden, First time mothers	Breast milk	1996-2012	-58.8
All Iceland, 3rd trimester	Maternal blood	1999-2004	-44,9
Costal Chukotka	Maternal blood	2001-2007	-19,0
Nunavik - pregnant Inuit	Maternal blood	1999-2013	-78,9
Reykjavik, 3rd trimester	Maternal blood	1995-2009	-51,2
Yup'ik	Maternal blood	1999-2012	13,6
Faroe Islands- Cohort 1	Children blood	2000-2009	-80,0
Faroe Islands- Cohort 3	Children blood	2002-2012	11,8
Nunavik	Children blood	2000-2007	-44,7
Northern Norway	Men blood	1979-2007	-76,5
Mirex			
Costal Chukotka	Maternal blood	2001-2007	-20,0
Nunavik - pregnant Inuit blood	Maternal blood	1999-2013	-76,9
Yup'ik	Maternal blood	1999-2012	-90,4
Nunavik	Children blood	2000-2007	-43,9
Northern Norway	Men blood	1979-2007	13,6
Oxychlordane			
All Iceland-pregnant blood 3rd trimester	Maternal blood	1999-2004	38,3
Costal Chuko	Maternal blood	2001-2007	-77,8
Disko Bay-pregnant Inuit	Maternal blood	1994-2006	-67,2
Nunavik-pregnant Inuit blood	Maternal blood	1999-2013	-71,4
Reykjavik-pregnant blood 3rd trimester	Maternal blood	1995-2009	-47,8
Yup'ik	Maternal blood	1999-2012	-74.8
Nunavik	Children blood	2000-2007	-40.0
Northern Norway	Men blood	1979-2007	-28,0
p,p'-DDT			
Costal Chukotka	maternal blood	2001-2007	-70,0
Disko Bay	maternal blood	1994-2006	-62,7
Nunavik	maternal blood	1999-2012	-83,1
Yup'ik	maternal blood	1999-2012	-60,9
Faroe Islands-Children Cohort 1	Children blood	2000-2009	-87,5
Faroe Islands-Children Cohort 3	Children blood	2000-2012	-90,4
Nunavik	Children blood	2000-2007	-42,3
Northern Norway	Men blood	1979-2007	-95,7
p,p'-DDE			
Sweden, first time mothers	Breast milk	1996-2012	-74,0
All Iceland-pregnant blood 3rd trimester	maternal blood	1999-2004	-46,0
Costal Chukotka	maternal blood	2001-2007	-70,4
Nunavik-pregnant Inuit	maternal blood	1999-2013	-79,7
Reykjavik-pregnant blood 3rd trimester	maternal blood	1995-2009	-68,1
Yup'ik	maternal blood	1999-2012	-38,7
Faroe Islands-Children Cohort 1	Children blood	2000-2009	-73,9
Faroe Islands-Children Cohort 3	Children blood	2000-2012	-84,9
Nunavik	Children blood	2000-2007	-41,4
Northern Norway	Men blood	1979-2007	-80,9

β-HCH			
All Iceland-pregnant 3rd trimester	Maternal blood	1999-2004	-62,5
Costal Chukotka	Maternal blood	2001-2007	-33,3
Nunavik-pregnant Inuit	Maternal blood	1999-2013	-80,0
Reykjavik-pregnant 3rd trimester	Maternal blood	1995-2009	-37,5
Yup'ik	Maternal blood	1999-2012	-46,3
Faroe Islands-Children Cohort 1	Children blood	2000-2009	-36,9
Faroe Islands-Children Cohort 3	Children blood	2002-2012	-88,9
Nunavik	Children blood	2000-2007	-53,0
cis-Nonachlor			
Nunavik-pregnant Inui	Maternal blood	1999-2013	-78,9
Northern Norway	Men blood	1979-2007	-8,3
trans-Nonachlor			
All Iceland-pregnant 3rd trimester	Maternal blood	1999-2004	-52,7
Costal Chukotka	Maternal blood	2001-2007	-77,8
Nunavik-pregnant Inuit	Maternal blood	1999-2013	-63,2
Reykjavik-pregnant 3rd trimester	Maternal blood	1995-2009	-44,2
Yup'ik	Maternal blood	1999-2012	86,8
Nunavik	Children blood	2000-2007	-28,6
Northern Norway	Men blood	1979-2007	-4,3
∑PCB			
Costal Chukotka	Maternal blood	2001-2007	-42,9
Faroe Islands-Children Cohort 1	Children blood	2000-2009	-71,0
Faroe Islands-Children Cohort 3	Children blood	2000-2012	-70,4
PCB28			
Sweden, First time mothers	Breast milk	1996-2010	-60,8
PCB99			
Nunavik	Children blood	2000-2007	-53.7
Northern Norway	Men blood	1979-2007	-65,8
PCB118			
All Iceland	Maternal blood	1999-2004	-21,4
Reykjavik-pregnant 3rd trimester	Maternal blood	1995-2009	-31,3
Yup'ik	Maternal blood	2004-2012	-39,3
Faroe Islands-Children Cohort 3	Children blood	2002-2012	-85,1
Nunavik	Children blood	2000-2007	-47,5
Northern Norway	Men blood	1979-2007	-60,0
PCB118			
All Iceland-pregnant 3rd trimester	Maternal blood	1999-2004	-42,5
Nunavik Inuit blood	Maternal blood	1999-2013	-82,7
Reykjavik-pregnant 3rd trimester	Maternal blood	1995-2009	-67,4
Yup'ik	Maternal blood	1999-2012	-74,0
Faroe Islands-Children Cohort 3	Children blood	2002-2012	-74,5
Nunavik	Children blood	2000-2007	-57,4
PCB138			
Central Finland, first time mothers	Breast milk	1987-2010	-82,3
Northern Finland, first time mothers	Breast milk	2005-2010	-1,6
Southern Finland, first time mothers	Breast milk	1987-2010	-80,5
Sweden, first time mothers	Breast milk	1996-2010	-67,6
All Iceland-pregnant 3rd trimester	Maternal blood	1999-2004	-33,3
Nunavik-pregnant Inuit	Maternal blood	1999-2013	-76,5
Reykjavik-pregnant 3rd trimester	Maternal blood	1995-2009	-50,0
Yup'ik	Maternal blood	1999-2012	-12,9

Faroe Islands-Children Cohort 3	Children blood	2002-2012	-66,5
Nunavik	Children blood	2000-2007	-46,4
Northern Norway	Men blood	1979-2007	-52,8
PCB180			
All Iceland-pregnant 3rd trimester	Maternal blood	1999-2004	-37,1
Nunavik-pregnant Inuit	Maternal blood	1999-2013	-81,1
Reykjavik-pregnant 3rd trimester	Maternal blood	1995-2009	-35,3
Yup'ik	Maternal blood	2004-2012	-46,0
Faroe Islands-Children Cohort 3	Children blood	2002-2012	-23,0
Nunavik	Children blood	2000-2007	-41,9
Northern Norway	Men blood	1979-2007	-38,1
Toxaphene Parlar26			
Nunavik	Maternal blood	2004-2013	-44,9
Northern Norway	Men blood	1979-2007	-57,8
Toxaphene Parlar50			
Nunavik	Maternal blood	2004-2013	-46,5
Northern Norway	Men blood	1979-2007	-47,1
PBDEs			
Central Finland, first time mothers	Breast milk	2000-2010	-48,4
Northern Finland, first time mothers	Breast milk	2000-2010	-36,0
Southern Finland, first time mothers	Breast milk	2005-2010	-55,2
PBDE47			
Sweden, first time mothers	Breast milk	1996-2012	-67,7
Yup'ik	Maternal blood	2004-2012	-23,8
PBDE99			
Yup'ik	Maternal blood	2004-2012	-28,6
PBDE100			
Yup'ik	Maternal blood	2004-2012	-13,2
PBDE153			
Sweden, first time mothers	Breast milk	1996-2012	42,1
Nunavik-pregnant inuit	Maternal blood	2004-2012	45,0
Yup'ik	Maternal blood	2004-2012	18,8
PFHxS			
Sweden, first time mothers	Breast milk	1996-2010	204.8
Northern Norway	Men blood	1979-2007	850.0
PFHxS			
Yup'ik	Maternal blood	2004-2012	75.0
PFOA			
Sweden- first-time mothers	Maternal blood	1996-2010	-34,6
Yup'ik	Maternal blood	2004-2012	233,3
Faroe Islands-Children Cohort 3	Children blood	2002-2012	-51,2
Northern Norway	Men blood	1979-2007	244,4
PFOS			
Sweden first-time mothers	Maternal blood	1996-2010	-72,5
Nunavik-pregnant Inuit	Maternal blood	2004-2012	-60,2
Yup'ik	Maternal blood	2004-2012	144,4
Faroe Islands-Children Cohort 3	Children blood	2002-2012	-60,5
Northern Norway	Men blood	1979-2007	283,7
Cd			
Yup'ik	Maternal blood	1999-2012	-33,3
Västerbotten	Men blood (25-35yrs)	1990-2009	0,0
Västerbotten	Men blood (50-60yrs)	1990-2009	0,0
Västerbotten	Women blood (25-35yrs)	1990-2009	-50,0

Västerbotten	Women blood (50-60yrs)	1990-2009	0,0
Total Hg			
Costal Chukotka	Maternal blood	2001-2007	0,0
Disko Bay-pregnant Inuit	Maternal blood	1994-2006	-14,3
Nunavik-pregnant Inuit	Maternal blood	1992-2013	-56,7
Yup'ik	Maternal blood	1999-2012	100,0
Västerbotten	Women blood (25-35yrs)	2004-2009	0,0
Västerbotten	Women blood (50-60yrs)	2004-2009	-7,1
Faroe Islands-Children Cohort 1	Children blood	2000-2009	-39,0
Faroe Islands-Children Cohort 3	Children blood	2002-2007	-23,1
Nunavik	Children blood	2000-2007	-45,8
Pb			
Costal Chukotka	Maternal blood	2001-2007	-21,1
Disko Bay-pregnant Inuit	Maternal blood	1994-2006	-50,0
Nunavik-pregnant Inuit	Maternal blood	1992-2013	-65,9
Yup'ik	Maternal blood	1999-2012	-32,7
Västerbotten	Men blood (25-35yrs)	1990-2009	-71,1
Västerbotten	Men blood (50-60yrs)	1990-2009	-57,2
Västerbotten	Women blood (25-35yrs)	1990-2009	-61,5
Västerbotten	Women blood (50-60yrs)	1990-2009	-47,5
Nunavik	Children blood	2000-2007	-46,3
