# Supplementary figures and images for: Temporal trends of contaminants in Arctic human populations
Source: Environ Sci Pollut Res Int. 2018 Aug 25;25(29):28834–50. doi: 10.1007/s11356-018-2936-8 (PMC6592971; doi:10.1007/s11356-018-2936-8)

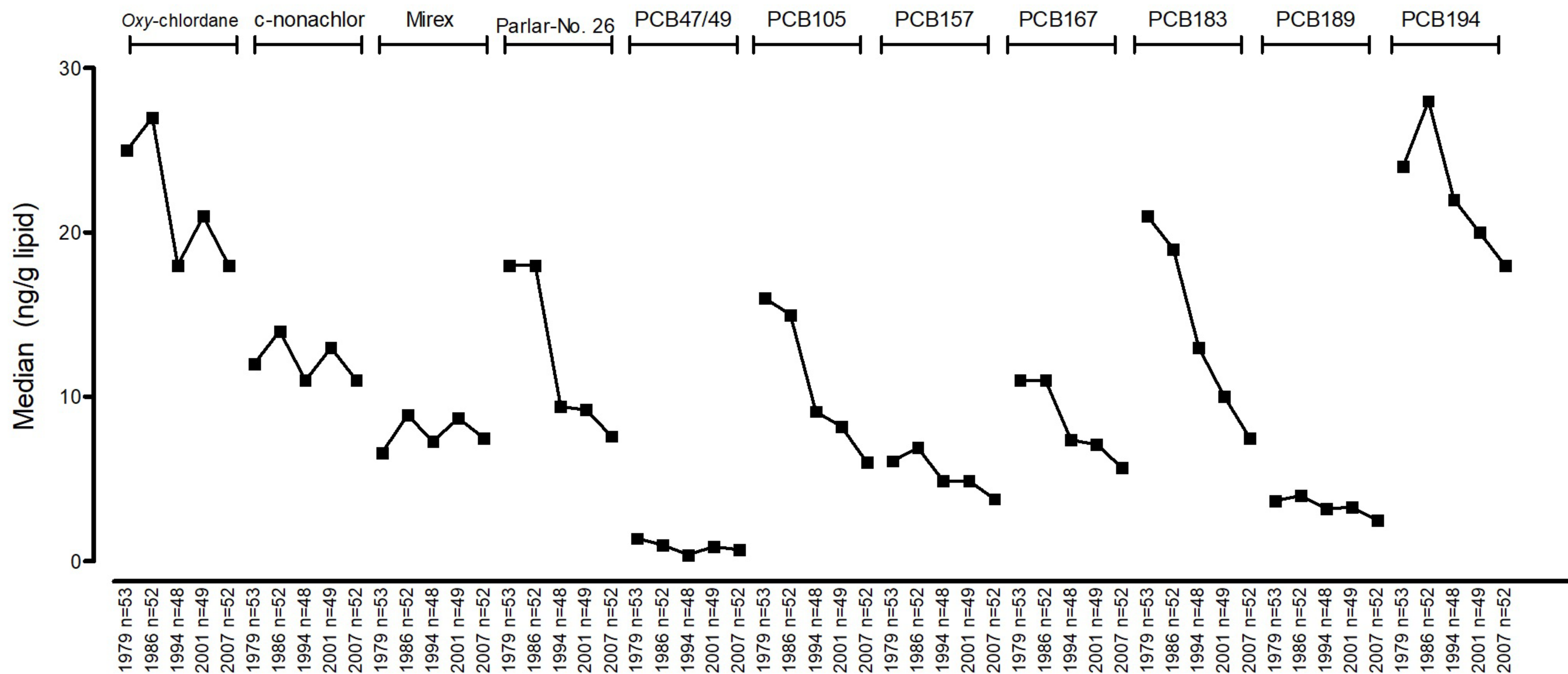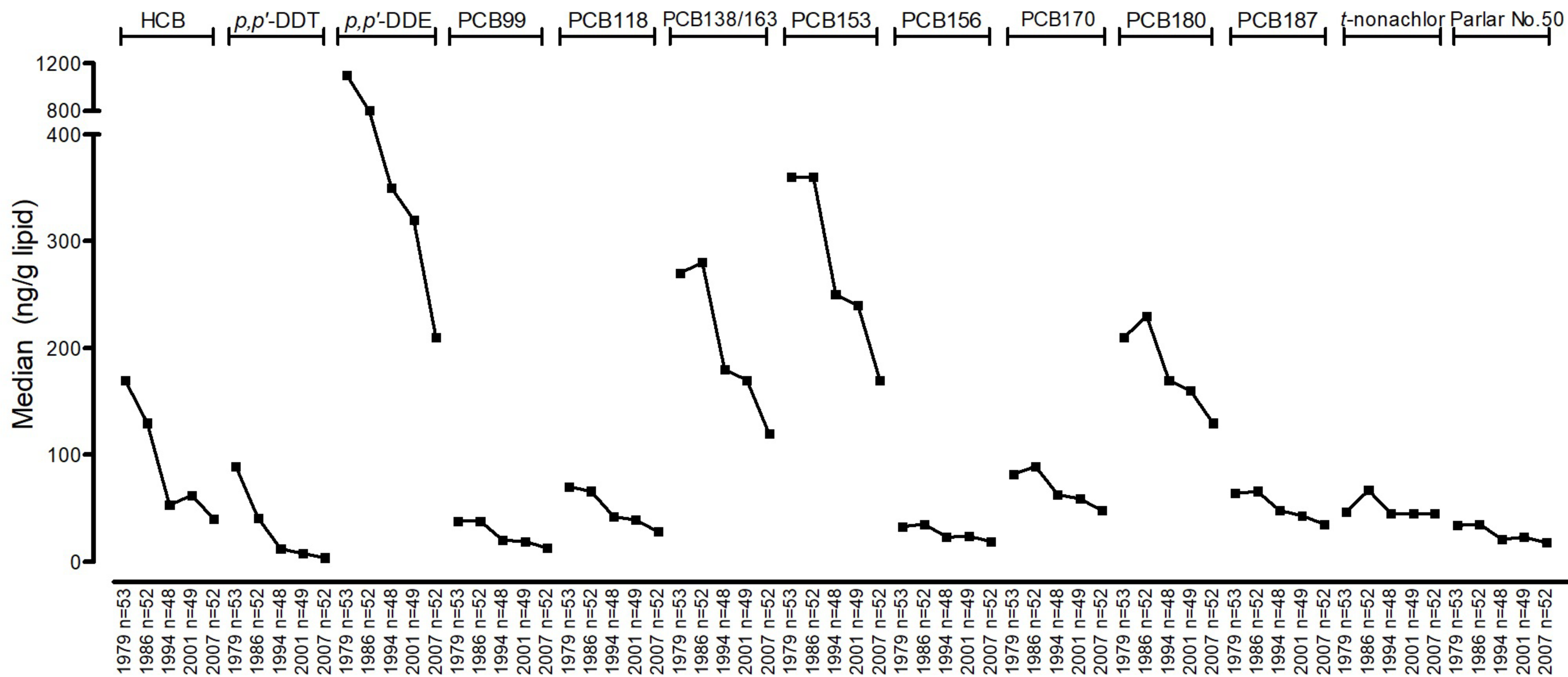

Supplement: Supplementary file 2 — Trends of contaminants concentrations (ng/g lipid) in serum samples of men in the Tromsø study (Nøst et al. 2014). (PDF 768 kb) [file 11356_2018_2936_MOESM2_ESM.pdf]
